# Improvement, Implementation, and Evaluation of the CMyLife Digital Care Platform: Participatory Action Research Approach

**DOI:** 10.2196/45259

**Published:** 2023-09-15

**Authors:** Lynn Verweij, Sanne J J P M Metsemakers, Geneviève I C G Ector, Peter Rademaker, Charlotte L Bekker, Bas van Vlijmen, Bert A van der Reijden, Nicole M A Blijlevens, Rosella P M G Hermens

**Affiliations:** 1 Department of Hematology Radboud University Medical Center Nijmegen Netherlands; 2 Department of Pharmacy Radboud University Medical Center Nijmegen Netherlands; 3 Laboratory of Hematology Department of Laboratory Medicine Radboud University Medical Center Nijmegen Netherlands; 4 Department of IQ Healthcare Radboud University Medical Center Nijmegen Netherlands

**Keywords:** eHealth, digital care platform, feasibility, patient experiences, usability, chronic myeloid leukemia, participatory action research, CMyLife

## Abstract

**Background:**

The evaluation of a continuously evolving eHealth tool in terms of improvement and implementation in daily practice is unclear. The CMyLife digital care platform provides patient-centered care by empowering patients with chronic myeloid leukemia, with a focus on making medication compliance insightful, discussable, and optimal, and achieving optimal control of the biomarker BCR-ABL1.

**Objective:**

The aim of this study was to investigate to what extent the participatory action research approach is suitable for the improvement and scientific evaluation of eHealth innovations in daily clinical practice (measured by user experiences) combined with the promotion of patient empowerment.

**Methods:**

The study used iterative cycles of planning, action, and reflection, whereby participants’ experiences (patients, health care providers, the CMyLife team, and app suppliers) with the platform determined next actions. Co-design workshops were the foundation of this cyclic process. Moreover, patients filled in 2 sets of questionnaires for assessing experiences with CMyLife, the actual use of the platform, and the influence of the platform after 3 and at least 6 months. Data collected during the workshops were analyzed using content analysis, which is often used for making a practical guide to action. Descriptive statistics were used to characterize the study population in terms of information related to chronic myeloid leukemia and sociodemographics, and to describe experiences with the CMyLife digital care platform and the actual use of this platform.

**Results:**

The co-design workshops provided insights that contributed to the improvement, implementation, and evaluation of CMyLife and empowered patients with chronic myeloid leukemia (for example, simplification of language, and improvement of the user friendliness of functionalities). The results of the questionnaires indicated that (1) the platform improved information provision on chronic myeloid leukemia in 67% (33/49) of patients, (2) the use of the medication app improved medication compliance in 42% (16/38) of patients, (3) the use of the guideline app improved guideline adherence in 44% (11/25) of patients, and (4) the use of the platform caused patients to feel more empowered.

**Conclusions:**

A participatory action research approach is suited to scientifically evaluate digital care platforms in daily clinical practice in terms of improvement, implementation, and patient empowerment. Systematic iterative evaluation of users’ needs and wishes is needed to keep care centered on patients and keep the innovation up-to-date and valuable for users.

## Introduction

The World Health Organization recognizes the challenges related to the growing burden of chronic diseases and cancer care [1]. The prevalence of patients with chronic diseases is increasing, a large shortage of health care professionals (HCPs) is predicted, and health care costs are rising [2-4]. Innovative approaches, like eHealth, could enable the delivery of sustainable, affordable, and patient-centered oncological care [1]. Therefore, a transition to patient-centered models of care in which cancer patients or survivors play active roles in the care process is needed for care to be future proof.

As part of this patient-centered approach, the improvement of patient empowerment is paramount [5,6]. The World Health Organization defines patient empowerment as “a process through which people gain greater control over decisions and actions affecting their health” [7]. Research has shown that innovations aimed at improving patient empowerment have a positive effect on care experiences and health outcomes, for example, more empowered patients have better treatment compliance, perform regular self-monitoring at home, and obtain more efficient care [6]. Besides patient empowerment, eHealth could also improve tailored information provision, enable the management of medication intake and adverse events more readily, and eventually decrease health care use by substituting regular care [8-12]. This shows that the benefits of eHealth are considerable and multidimensional [13].

The implementation of eHealth, particularly digital care platforms, is deemed difficult, and progress in this field is accelerating [8,12-14]. Studies agree that systematic iterative evaluation of such innovations needs to be continued after development [13-15]. Moreover, studies have shown that involving stakeholders during the process facilitates implementation [12]. The information presented by eHealth innovations needs to be up-to-date or else the innovations will lose value and credibility among HCPs and patients [14]. It is crucial to show the evidence-based added value of innovations in order to keep HCPs and patients interested in sustained use [12,16,17]. The question is which scientific method could best serve the purpose of evaluating the effectiveness, feasibility, and usefulness of such an evolving tool in daily clinical practice. Participatory action research (PAR) seems to suit this purpose since improvement and evaluation go hand-in-hand. PAR is defined by Baum et al [18] as research that seeks to understand and improve the world by changing it. It includes iterative cycles of planning, action, and reflection, whereby participants collect and analyze data, and determine what actions should follow. The process of PAR should empower participants and lead to increased self-control [18]. PAR helps to gather feedback relatively quickly about what works and what does not work. However, the PAR approach for eHealth in the field of oncology has been rarely described in the literature. Our study is the first to evaluate and improve a patient-centered digital care platform, using the PAR approach.

The CMyLife digital care platform provides patient-centered care by empowering patients with chronic myeloid leukemia (CML) [12,19-21]. CML is a hematological malignancy caused by a chromosomal translocation that gives rise to constitutively active tyrosine kinase protein (BCR-ABL1) [22]. Since the advent of orally administered tyrosine kinase inhibitors (TKIs), the survival of patients with CML has been similar to that of the general population, provided that patients have an optimal response to the treatment [23]. Medication compliance and guideline adherence are 2 determining factors of the achievement of an optimal response to treatment. CMyLife aims to provide CML patients with tools and knowledge to take a more active role in their care process and improve their medication compliance and molecular monitoring, which could ultimately lead to an increased quality of life and the opportunity of more remote care. The main parts of CMyLife are a website, apps to monitor medication compliance and control of biomarker levels, and a personal health environment. The first version of CMyLife, developed in close cooperation with patients using a design thinking approach, was launched in 2016, and it has been freely accessible ever since [19]. However, as mentioned above, the evaluation of such a continuously evolving tool in daily clinical practice is deemed challenging but important.

Our PAR involved patients with CML in the effort to improve their own health, with a focus on making medication compliance insightful and discussable for shared decision-making and improvement, and achieving optimal control of biomarker levels (guideline adherence). We studied to what extent the PAR approach is suitable for the improvement and scientific evaluation of eHealth innovations in daily clinical practice (measured by user experiences) combined with the promotion of patient empowerment.

## Methods

### Design

A PAR design was used to improve, implement, and evaluate the CMyLife digital care platform and promote patient empowerment. The study was performed from May 2018 to October 2020, and used iterative cycles of planning, action, and reflection, whereby participants’ experiences with the platform determined actions. Depending on the input of users and interim results, the aims, research methods, and actions were adjusted.

### Ethical Considerations

Given the nature of this study and the low impact on study participants, the Medical Research Involving Human Subjects Act (Dutch: Wet medisch-wetenschappelijk onderzoek met mensen) does not apply, as confirmed by the Institutional Medical Ethics Committee “CMO Regio Arnhem-Nijmegen” (dossier number: 516006001). All participants signed an informed consent form before participation in the study. The Medical Research Involving Human Subjects Act does not apply and this research has a low intensity/impact on participants for the following reasons: (1) This research did not subject participants to actions, and rules of conduct were not imposed on them; (2) Participants varied between questionnaires and workshops, and it was not the case that 1 participant repeatedly participated in the workshops and questionnaires; (3) The questions in the questionnaires or during the co-design work were not difficult or intrusive, they were not emotionally or psychologically burdensome, and they were simple and easy to respond to; (4) The questionnaires did not take a long time to fill in; and (5) Participation was voluntary, and participants signed an informed consent form. Multimedia Appendix 1 and 2 present the appropriate checklists for the study design.

### Setting

In the Netherlands, CML treatment is organized in 8 academic hospitals and 68 smaller nonacademic hospitals. Patients’ biomarker levels are monitored during their entire life. The latest evidence-based CML guidelines recommend that the molecular treatment response should be measured at least every 3 months in the first year after diagnosis and every 4 to 6 months thereafter [24]. Multimedia Appendix 3 shows the key points of the CML guidelines. Only medical specialists (eg, hematologists) can prescribe CML medication according to the Dutch expensive medicine regulation. Health care insurance, which covers all CML care, is mandatory in the Dutch health care system; hence, treatment is accessible to all patients. Treatment results differ between hospitals with high and low numbers of CML patients receiving treatment because CML is a rare disease [25]. The CMyLife digital care platform, developed using a design thinking methodology, aims to provide CML patients with tools and knowledge to self-monitor their disease process, interpret it, and act on it [19]. The CMyLife digital care platform is described below.

### Intervention

CMyLife consists of multiple features comprising a website [26], a personal health environment, and 2 apps to monitor medication compliance and guideline adherence, called the medication app and the guideline app. All data are secured and conform to the General Data Protection Regulation and the Dutch security guideline (NEN7510). Features containing data from the electronic medical records (EMRs) of hospitals (ie, biomarker levels) are secured with a double-authentication procedure. The platform stimulates patients to get their medication delivered at home and perform local blood tests. The *website* provides accurate and easy-to-understand information about living with CML (medication, guidelines, side effects, and the effect on daily life). The website enables patients to communicate with specialists and other patients through a forum. The *personal health environment* consists of a Patient Knows Best portal [27]. Patients can save their medical records, for example, side effects, from their EMRs in their personal health environment and share this with their HCPs. The medication app [28] is used to improve medication compliance. The app aims to make patients’ medication compliance insightful, and easy to share and discuss with their HCPs. It is also possible to set medication reminders, request for repeat medication prescriptions, read the information leaflet of the medication, and log side effects, which can be shared with HCPs through the personal health environment. To achieve optimal monitoring of the biomarker BCR-ABL1 (guideline adherence), the guideline app [24] was developed with patients. This app sends reminders when it is time for patients to undergo biomarker level assessment in accordance with the Dutch CML guidelines. It also shows a visual and understandable explanation of BCR-ABL1 levels in relation to the Dutch guidelines. Therefore, this app gives patients control over their own guideline adherence and enables them to contact their HCPs if treatment goals are not reached.

### Study Population

**Figure 1:**
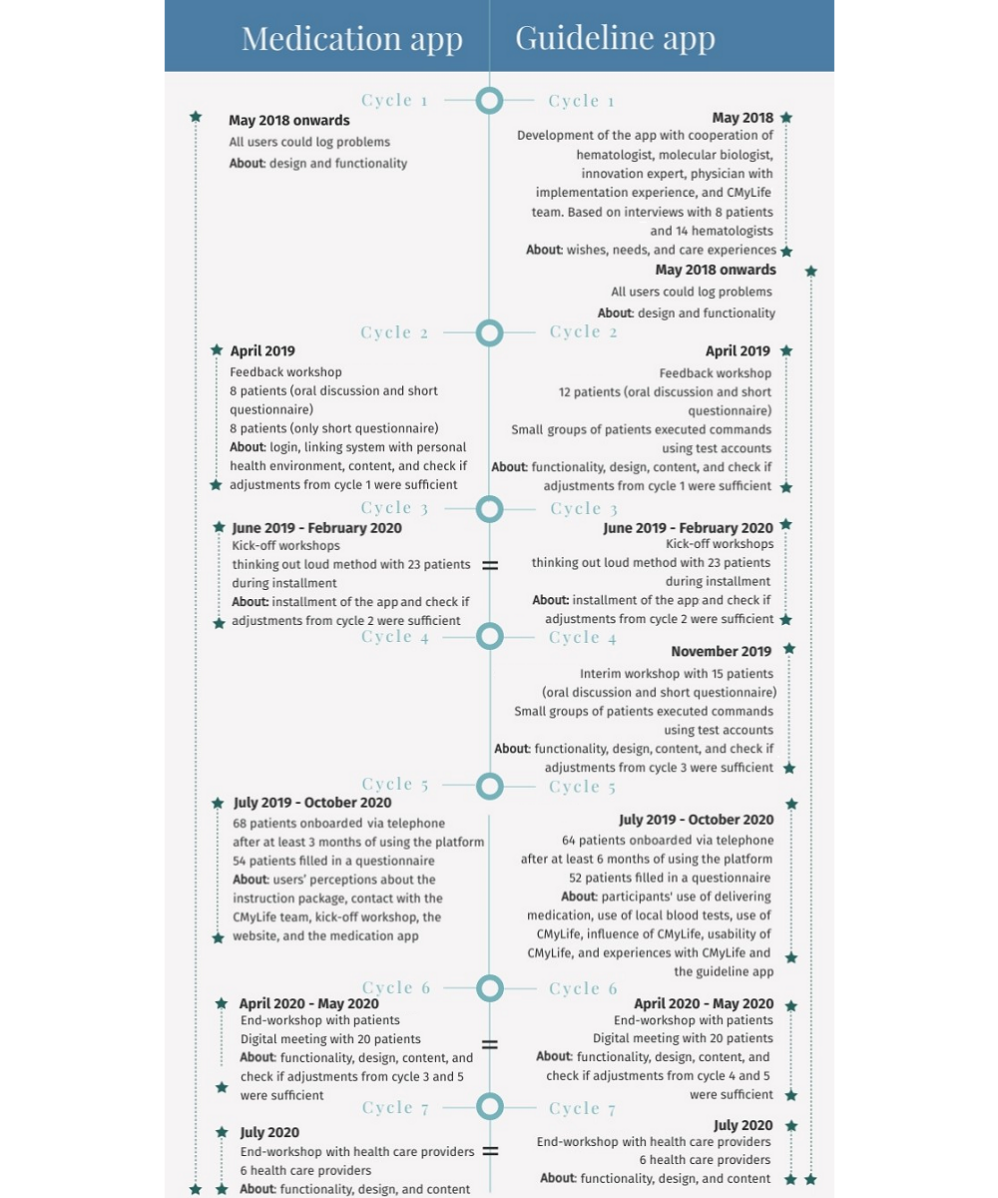
Description of the iterative cyclic process of the participatory action research approach.

During this study, patients, HCPs, the CMyLife team (responsible for supporting users of the digital care platform), and app suppliers were involved in the entire process. Included patients were in the chronic phase of CML and were treated with first- or second-line TKIs. First-line TKIs were the first TKIs initiated. If the response to the treatment was inadequate or patients were intolerant, they were switched to a subsequent TKI (eg, second-line of subsequent-line TKIs). Patients who were treated with second-line TKIs were only allowed to participate if they had shifted from first-line to second-line TKIs as a result of intolerance (not because of treatment failure). Patients in treatment-free remission, those in the acceleration phase, those experiencing blast crisis, and those planning pregnancy during the study period were excluded from this study as these factors could influence the study results. Patients were enrolled from 20 different hospitals spread across the Netherlands (5 academic hospitals and 15 nonacademic hospitals) via their HCPs, the CMyLife website, or the patient advocacy group Hematon. HCPs were enrolled from 12 different hospitals spread across the Netherlands (5 academic hospitals and 7 nonacademic hospitals). The numbers of patients and HCPs per cycle are shown in Figure 1. The CMyLife team included 4 project staff members (who were in continuous contact with patients, HCPs, and app suppliers), the CMyLife program manager, a physician with eHealth expertise, a patient, a specialized nurse, a researcher, and a research assistant. Patients were supported in using CMyLife by an instruction package and a kick-off workshop. Patients and HCPs were provided with adequate support in using CMyLife by the CMyLife team, and they were informed about when to use the platform and the benefits of using it. The CMyLife team was reachable for questions during the day and offered help to all users for installing the platform. Moreover, the CMyLife team gathered feedback from users during workshops. App suppliers had to meet the General Data Protection Regulations and Medical Devices Regulations, and had to work in accordance with the Dutch security guideline (NEN7510). They were in continuous contact with the CMyLife team for improving the apps.

### Data Collection

Further development of the CMyLife platform was performed using an iterative cyclic process, including co-design workshops and questionnaires (described in Figure 1). The co-design workshops were the foundation of the iterative process. Additionally, patients using the CMyLife platform filled in 2 sets of questionnaires. The medication app was an already existing app; therefore, the aim was to further develop and adjust it to the wishes and needs of patients with CML. The guideline app was developed entirely for patients with CML during this PAR.

### Co-design Workshops

A total of 3 types of workshops were planned based on co-design activity methods, including feedback workshops, kick-off workshops, and end workshops. The experiences of patients were collected during the co-design workshops, including testing of the apps, oral discussions, short questionnaires, and focus group interviews. The process of walking through the experiences of participants with the apps helped to guide and focus improvements and adjustments of the platform [29]. The results of the workshops provided input for the actions that should be undertaken. When determining new actions, patients, HCPs, and the CMyLife team were involved and eventually app suppliers were consulted. Workshops were facilitated by members of the CMyLife team. All discussions were audio recorded, and notes were collected with the consent of all workshop participants. The facilitators of the workshops used semistructured guides for each workshop to guide the discussion. The guides were used to ensure the overall sense of direction throughout the activities, but participants had sufficient freedom to expand and ask for additional clarification on specific topics and thereby gain in-depth understanding. Participants were divided into smaller groups during the workshops. The use of smaller groups ensured that all participants were able to contribute [30,31]. All workshops included a short questionnaire about the use and quality of the apps, which gave participants, who were uncomfortable discussing topics in a group setting, the possibility to provide feedback. Moreover, participants were asked about the quality of the workshops on a scale from 1 to 5 (a higher score indicated better quality).

### Questionnaires

After 3 and 6 months of using CMyLife, participants filled in the first and second questionnaires, respectively. The first questionnaire contained questions about patient characteristics, user perception of the instruction package, contact with the CMyLife team, the kick-off workshop, the website, and the medication app. The second questionnaire contained questions about use of medication delivery, use of local blood tests, use of CMyLife, user perception of the influence of CMyLife (several aspects, including patient empowerment, medication compliance, guideline adherence, and information provision), usability of CMyLife, and experiences with CMyLife and the guideline app. The second questionnaire was more extensive than the first because patients then had more experience with the different components of CMyLife. Moreover, the first questionnaire focused on the medication app since this was an already existing app, and the second questionnaire focused on the guideline app since patients needed time to use the newest version of the app. The System Usability Scale (SUS) was used to measure the usability of the apps [32].

### Analysis

Data collected during the workshops were analyzed using content analysis, which is often used for making a practical guide to action [33]. During the workshops, the content of the apps was discussed orally and questionnaires were filled in. The results were analyzed by researchers and discussed with the CMyLife team and app suppliers to improve the apps. All measurements of the questionnaires were processed anonymously, and analyses were performed using SPSS version 25 (IBM Corp). Descriptive statistics were used to characterize the study population in terms of CML-related information and sociodemographics, and to describe experiences with the CMyLife digital care platform and the use of this platform. The questionnaires contained several kinds of questions and answers. The Likert scale was mostly used for measuring attitudes, perceptions, and opinions about the features of the CMyLife platform [34]. The features of CMyLife were graded on a scale from 1 to 10 (not validated, but a conventional method in the Netherlands). To measure the influence of the features on certain concepts, the top 3 different features of CMyLife were asked, and the values were converted to sum scores, with a higher sum score indicating more influence on the mentioned aspect. This allowed researchers to compare the features properly. The SUS scores ranged from 0 to 100. The SUS is a validated and widely used questionnaire to measure usability [32,35]. A SUS score of <51 indicates poor usability, 51-68 indicates acceptable usability, 68-80.3 indicates good usability, and 80.3-100 indicates excellent usability.

## Results

### Participant Characteristics: Iterative Cycles

Patients, HCPs, the CMyLife team, and app suppliers were involved during this PAR. The included patients were diverse, and gender, age, first- or second-line TKIs, years since diagnosis, digital skills, and education varied (characteristics are mentioned below). The numbers of participants for each cycle are depicted in Figure 1. The results of the PAR are described below for the different components of the CMyLife platform. The results show that the iterative cycles of planning, action, and reflection led to the evaluation of participants’ experiences with the CMyLife digital care platform, which eventually determined actions that improved the platform, particularly regarding content and the functionality of features.

### Patient Characteristics: Questionnaires

**Figure 2:**
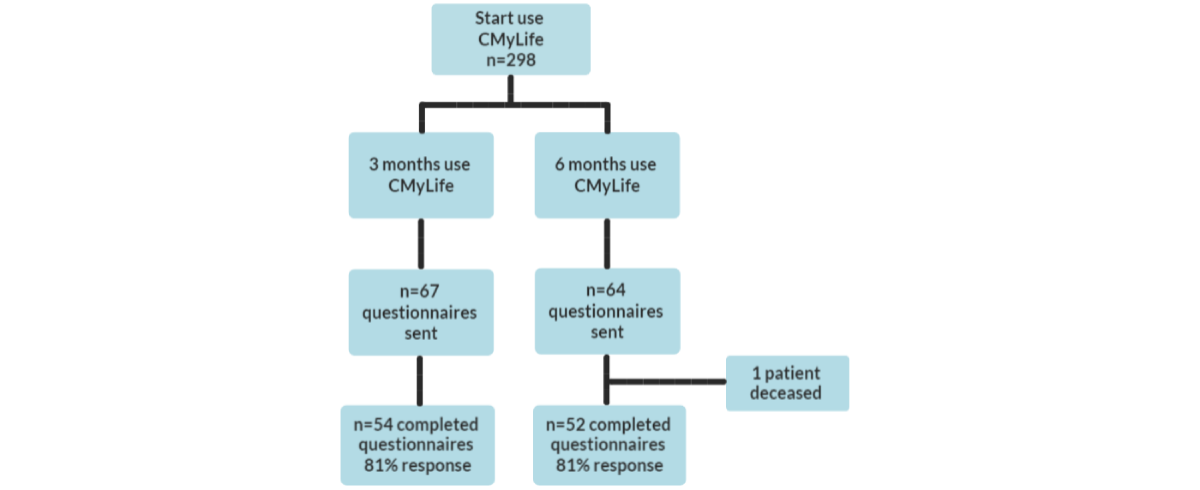
Flowchart of questionnaires.

After 3 months of using CMyLife, 67 questionnaires were sent, of which 54 were completed (81% response rate). After 6 months of using the platform, 64 questionnaires were sent, of which 52 were completed (1 patient died; 81% response rate) and 3 were partially completed. Figure 2 shows the flowchart of the questionnaires during the PAR. Table 1 summarizes the patient characteristics. The majority of users were male (after 3 months: 35/53, 66%; after 6 months: 33/52, 63%), were aged below 65 years (after 3 months: 34/52, 65%; after 6 months: 35/51, 69%), and used CMyLife before the study period (after 3 months: 30/51, 59%; after 6 months: 33/50, 66%).

**Table 1:** Baseline characteristics of patients with chronic myeloid leukemia who filled in the study questionnaires after 3 and 6 months of using CMyLife.

Characteristic		After 3 months of using CMyLife (n=54)	After 6 months of using CMyLife (n=52)
**Gender, n (%)**			
	Male	35 (65)	33 (63)
	Female	18 (33)	19 (37)
	Missing	1 (2)	0 (0)
**Age (years), n (%)**			
	18-64	34 (63)	35 (67)
	65 or older	18 (33)	16 (31)
	Missing	2 (4)	1 (2)
**Education level^a^, n (%)**			
	High	25 (46)	28 (54)
	Middle	23 (43)	18 (35)
	Low	5 (9)	6 (11)
	Missing	1 (2)	0 (0)
**Years since diagnosis, mean (SD)**		5.5 (5.7)	5.9 (6.4)
**Currently using a TKI^b^, n (%)**			
	Yes, imatinib	30 (55)	31 (60)
	Yes, others^c^	22 (41)	20 (38)
	Missing	2 (4)	1 (2)
**Previously treated with a TKI, n (%)**			
	Yes	19 (35)	16 (31)
	No	28 (52)	29 (56)
	Missing	7 (13)	7 (13)

^a^High education level included higher vocational education and academic education; middle education level included secondary vocational education, higher general secondary education, and preuniversity education; low education level included primary education, lower general secondary education, and preparatory secondary vocational education.

^b^TKI: tyrosine kinase inhibitor.

^c^Mostly dasatinib and nilotinib.

### Instruction Package

All results from the first questionnaire are shown in Multimedia Appendix 4. The most prominent and relevant results are described in the text below. The results showed that 80% (43/54) of patients found the instruction package very usable and 82% (44/54) found it very understandable. The apps were successfully installed by 82% (44/54) of patients with the help of the instruction package or the CMyLife team. Patients provided a score of 7.1 out of 10 for the instruction package. The instruction package was clear and complete, and the explanation and support were good. In addition to the positive points of the components evaluated, suggestions were also gathered. The language of the instruction package was still too difficult for some patients, and they would have liked it to be simpler. Moreover, they indicated that a version for Android or Apple would be better since there was only a single general version that was not fit for all devices. Lastly, an English version of the instruction package would be an improvement since the first version was only in Dutch. These aspects were taken into account, and the instruction package was improved accordingly. The text was simplified, and pictures of the steps were added. Moreover, an English version was developed, and the instruction package was made fit for all devices. At the end of this study, all patients who wanted to use the CMyLife platform received the improved instruction package. Some participants commented as follows:

It would be helpful if there were pictures with each step of installing the apps.

Use less formal language, make it more simple.

### Contact With the CMyLife Team

In addition, 93% (50/54) of patients thought contact with the CMyLife team for installing the apps was very pleasant. Patients mentioned that contact with the CMyLife team was result-oriented, and time was not an issue. Members of the CMyLife team were reported to be very helpful, friendly, patient, and knowledgeable. Suggestions for contact with the CMyLife team included contact at night and more frequent initiatives to check the situation. Patients mentioned that they would have liked it if there was more room for personal attention during the workshops and they desired to know more clearly what the CMyLife team wanted to know from users. The CMyLife team took these improvement points into consideration, and they will try to take them into account in the future to improve contact with the team according to the needs of patients. They functioned more like providers of personal logistics and assistance (a sort of help desk). Examples of improvements were as follows: send participants an email with information or call them about the goals and planning of the co-design workshops before they participate, check in with patients more often to assess their situation and ask if everything is clear and if they need anything from the team, and plan more time for the co-design workshops to enhance personal attention. Some participants commented as follows:

Helpful, friendly, and patient.

I work during the day, therefore, contact at night would be better.

### Kick-off Workshops

Positive points mentioned by participants about the kick-off workshops were as follows: presence of a hematologist, contact with fellow patients, informativeness, usefulness, and a small setup. Moreover, 93% (26/28) of patients who attended the kick-off workshops reported that they thought the workshops were very informative. The mean score of the kick-off workshops was 8.2 out of 10. Some participants commented as follows:

The presence of a hematologist is great.

Definitely in the test phase, take some more time for these workshops to be able to have some personal contact.

It would be a good idea that the person who will have personal contact with patients is present at these workshops.

### Website

In 2018, the website had 9159 visitors and 472 registered accounts. In 2019, this increased to 14,448 visitors and 625 registered accounts. Regarding the use of the website, information about the disease was used by 74% (28/38) of patients. All patients would recommend the website to other people with CML. In addition, 97% (37/38) of patients agreed that the language used was very understandable. Patients liked to see more information about research, pregnancy, dealing with side effects, and stopping treatment on the website. In particular, new CML patients liked that there is a website with reliable, clear, and comprehensive information focused on patients with CML, including actual topics like COVID-19 and summaries of medications and their side effects. They liked the forum for reading the stories of fellow patients. In contrast, some patients reported becoming anxious and worried by the forum. In addition, patients who were diagnosed with CML for a long period and had a stable condition were less interested in using CMyLife compared with newly diagnosed patients. The desires of patients are continuously being evaluated and added to the platform. Since there were some contradictory results, it was difficult to improve these aspects. The platform tries to provide all desired tools and knowledge in an understandable way, and patients can decide the most appropriate use themselves. In this way, the platform can be personalized by users. For example, patients who are stable for a long period can only use information about stopping treatment and can skip information about the disease. Some participants commented as follows:

I don’t see the added value of using the website for me, I am used to living with the disease and stable.

It is nice to stay up to date with the news on the website.

Make the website more understandable for older people.

### Medication App

The medication app was used by 72% (39/54) of patients who filled in the first questionnaire, of which 64% (25/39) used the app daily. With 85% (33/39) of patients logging medication intake moments, this was the most used component of the medication app. Moreover, 77% (30/39) of patients reported that they would recommend using the medication app to other people with CML. The reminder function, simplicity and clarity of the app, and inventory management were mentioned as positive points of the medication app. During co-design workshops, participants had some suggestions for improvement as follows: incorporation of blood values, connection between the health app from Apple and the medication app, connection with the hospital, reduction of the frequency of logging side effects, and optimal functioning of the diary. Some patients did not see the added value of the medication app and reported that the menu structure was not clear. Patients would have liked to see their medication compliance over a longer period in the app. Moreover, some side effects were missing (mental complaints, dizziness, and neuropathy in the feet and hands), and some functionalities did not work optimally. These suggestions were transformed into actions and taken into account in the further improvement of the app, for example, side effects were added, the frequency of logging side effects was lowered, and functionalities were improved. One participant commented as follows:

Receiving a reminder for registering side effects only once a week would be better. Like, take a look back at your week which side effects did you suffer from, I would actually do that.

### Medication Delivery and Local Blood Tests

All results from the second questionnaire are shown in Multimedia Appendix 5. The most prominent and relevant results are described below. Half (26/52, 50%) of the patients had their medications delivered at home, of which 23% (6/26) had their medications delivered via the medication app. Blood was drawn at the treating hospital in 83% (43/52) of patients.

### CMyLife Use and Features

Only 6% (3/52) of patients did not use the CMyLife platform in the past 6 months. Patients indicated that information on the CMyLife website had the most positive influence on their knowledge about CML, degree of control over their disease, and insights regarding side effects and complaints. The SUS showed mean scores of 65.3 and 60.0 for the medication app and guideline app, respectively.

The results showed that 48% (23/48) of patients learned something new about the disease because of using CMyLife, for example, about heredity, the course of the disease, the origin of the disease, experiences with diverse medication, and the effects of COVID-19. In addition, 46% (22/48) of patients learned something new about treatment of the disease because of using CMyLife, for example, medication mechanism of action, research and new medications, treatment-free remission, and switch in TKIs. Moreover, 35% (17/48) of patients learned something new about side effects and complaints because of using CMyLife. Some examples of new information were not being the only one experiencing certain side effects, details of how others deal with side effects, and experiences of other patients.

**Figure 3:**
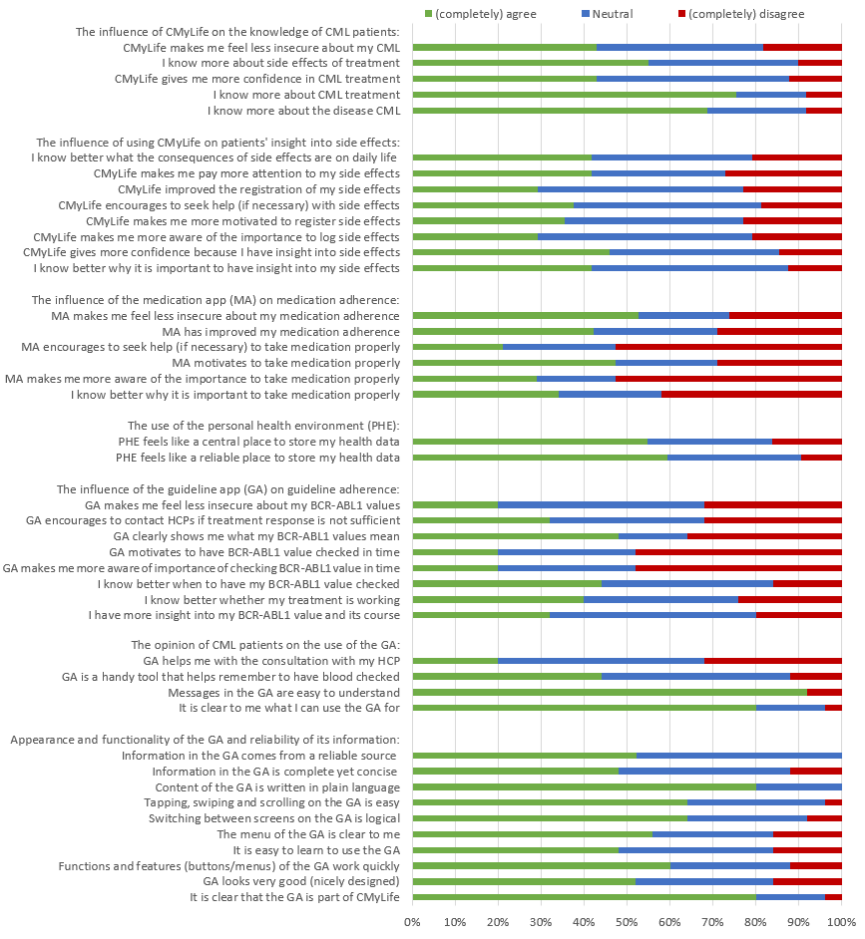
Percentages of patients who completely agreed with, completely disagreed with, or were neutral toward statements about the influence, use, and functionality of features of CMyLife. CML: chronic myeloid leukemia; GA: guideline app; HCP: health care professional; MA: medication app; PHE: personal health environment.

Figure 3 shows the percentages of patients who completely agreed with, completely disagreed with, or were neutral toward statements about the influence, use, and functionality of the features of CMyLife. Knowledge about the disease and treatment increased in 67% (33/49) and 76% (37/49) of patients, respectively. CMyLife made 46% (22/48) of patients more confident because they gained insights about their side effects, and the use of the medication app made 53% (20/38) of patients feel less insecure about their medication compliance. Moreover, 42% (16/38) of patients indicated that their medication compliance improved when using the medication app. The personal health environment felt like a reliable and central place to store personal health data for 59% (19/32) and 55% (17/31) of patients, respectively. Furthermore, 44% (11/25) of patients reported that they knew better when to get BCR-ABL1 values checked, and that the guideline app is a handy tool for this purpose. Logging side effects and entering medication in the personal health environment were very easy for 42% (14/33) of patients, and entering BCR-ABL1 values in the personal health environment was very easy for 30% (10/33) of patients. Linking the guideline app and the medication app to the personal health environment was very easy for 39% (13/33) and 24% (8/33) of patients, respectively.

### Guideline App

During this PAR, the guideline app was developed, evaluated, improved, and used. Among all patients who filled in the second questionnaire after at least 6 months of using CMyLife, 48% (25/52) used the guideline app. In addition, 36% (9/25) of these patients used the app monthly and 84% (21/25) would recommend use of the app to other people with CML. The positive points of the guideline app were simplicity and clarity of the app, clear messages, confidence boosting, insights about BCR-ABL1 values, overview of the disease course, and information about the next blood examination. Linking the guideline app with other apps was suggested by several patients. Some patients also mentioned that the guideline app should be more user friendly, and it was mentioned that sometimes the app was not up-to-date. These suggestions were taken into account in the further improvement of the app. Some examples of actions that led to improvements are as follows: ensuring that the app is up-to-date, improving understandability of the text, and making the app more user friendly. Some participants commented as follows:

Connection between the apps and the hospital is a must.

There is too much text, make it more clear and make it more user-friendly.

The guideline app gives insight in disease and treatment in addition to the information from your specialist.

## Discussion

### General Findings

The results showed that the iterative cycles of planning, action, and reflection, whereby participants’ experiences with the real-life digital care platform CMyLife determined actions, helped to evaluate, improve, and implement the platform. Moreover, the results showed that the platform improved patients’ knowledge about their disease and its treatment, use of the medication app improved patients’ medication compliance, patients knew better when to have their BCR-ABL1 values checked, and patients felt more empowered when using the platform. Therefore, PAR is suited for the improvement and evaluation of eHealth innovations in daily clinical practice, which goes hand-in-hand with promoting patient empowerment since patients feel more empowered.

### Comparison With Previous Research

eHealth innovations play a substantial role in shaping health care systems, but until innovations are “fit for purpose,” the risk of failing implementation is high, and the challenges of evaluating these complex interventions have been recognized [13,36]. Several studies suggested that to gain the most from eHealth innovations, it is important to match the innovation with what is needed in practice, to work together with relevant stakeholders in all stages of the project, and to perform continuous systematic evaluations of the evolving tools [13-15,37]. However, it is difficult to choose a proper methodology that serves these purposes. A randomized controlled trail, which is the golden standard of testing a hypothesis, cannot be used to evaluate eHealth since it is simply not possible to blind patients regarding participation in the intervention group (using eHealth) or the control group (not using eHealth). Moreover, it would not be ethical to withhold patients from using something that benefits their health.

PAR seems to suit the purpose of bridging the gap between basic scientific methods and clinical practice [38]. Action research not only produces knowledge, like conventional quantitative and qualitative research methodologies, but also combines it with action and reflection cycles [39]. PAR focuses on involving users during the action research [40]. A review by Oberschmidt et al [41] aimed to provide more knowledge on the best practices and lessons learned from action research studies in eHealth. The review focused on a variety of eHealth innovations, ranging from EMRs, health information systems, and mental health services to telemonitoring and health promotion and education, which differ a lot from our multicomponent digital care platform [41]. Moreover, the target groups of the included studies varied. Only 3 studies focused on patients with cancer. Despite the variations in the target groups and included eHealth innovations, the authors stated that their recommendations are not exclusively related to eHealth and may be relevant for other fields. Their most important recommendations include paying attention to training regarding stakeholders’ skills and confidence, and the different roles and tasks of researchers. They also highlighted the need for frequent reflection and understandable dissemination suiting the target group. Therefore, the target group should be involved and informed properly about the innovation and the research.

In our study, participants were provided with an instruction package, which helped them to use the platform. Moreover, during the workshops, patients were guided and trained by the CMyLife team in using the platform and evaluating it. Attention was not necessarily paid to the different roles of researchers as described by Oberschmidt et al [41], for example, fostering a welcoming environment for all stakeholders was paid attention to by the CMyLife team. Therefore, this did not limit our study. Furthermore, frequent reflections of the changes of previous cycles, the process, and the current status of the platform were accurately performed as depicted in Figure 1. Finally, Oberschmidt et al [41] reported that researchers of action research need to communicate findings to the academic world and inform the target group about the project in ways that suit users’ needs. In our study, researchers communicated the findings to the academic world, while the CMyLife team informed the target group.

### Strengths and Limitations

A PAR approach has several strengths for the evaluation, improvement, and implementation of the real-life digital care platform CMyLife and for empowering patients with CML. It is an approach unique to a particular context as it revolves around the wishes and needs within a particular group of people in daily clinical practice. Our PAR aimed to improve empowerment as well as evaluate it [42]. End users of the CMyLife platform have been involved in not only the development and implementation of CMyLife, but also each step of the improvement process to establish true patient-centered care. End users provided unique perspectives based on their own experiences [43]. PAR allows the evaluation of constantly evolving innovations. This PAR involved both qualitative (eg, interviews) and quantitative (eg, questionnaires) research methods to evaluate the CMyLife digital care platform, which is another strength of this study.

Regarding the limitations of our PAR approach, participant commitment is the foundation on which PAR can emerge. It requires participants to dedicate time, to be open minded, to accept a degree of uncertainty, and to be interested in the process. All our study participants were willing to use CMyLife, and patients with low literacy were absent. This may have overestimated the results of the questionnaires, limiting generalizability to the larger population of patients with CML. The literature shows that patients with low literacy are difficult to involve in eHealth research [44]. It is a possibility that the CMyLife platform requires too high literacy levels. The CMyLife team will continue to try and reach this group of patients with the platform through ongoing qualitative studies in cooperation with communication and information sciences students for evaluating the needs of these patients, improving the CMyLife platform, and making it more accessible to patients with low literacy. It is important to connect with them through people they trust, for example, their hematologist or a nurse. To further improve the platform and reduce inequalities, CMyLife takes advise from Pharos, which is the national center of expertise on health disparities [45].

### Future Perspectives and Research Directions

It is important for the systematic iterative evaluation of eHealth innovations, like CMyLife, to be continued after development and implementation [13-15]. Moreover, such innovations should be continuously adapted to keep information up-to-date. Without these continuous evaluations of the innovation, it will lose value and credibility among clinicians and patients [14]. CMyLife included users from the beginning of the project and will never stop involving them in the improvement, and the platform will continue to be evaluated, adapted, and improved. The medication app and the guideline app scored 65.3 and 60.0 points, respectively, on the SUS. This shows that the apps are acceptable, and there is still room for improvement. To validate whether the apps improve, the SUS scores will be evaluated in the future. This study describes the first period of the platform from May 2018 to October 2020, and the platform was improved a lot at the end of this study period compared to the beginning. Eventually, patient-reported information, which is automatically gathered through the digital care platform (for example, routine gathering of patient-reported outcome measures or treatment outcomes), can be used for guideline development. Involving data derived from patient-reported outcome measures in the guideline development process could help to create personalized guideline development [46-48]. The use of aggregated patient-reported outcome data might be able to fill in knowledge gaps in the current guideline and provide more diverse real-life data of a longer follow-up. In addition, it could add the patient perspective, and thereby complement and benchmark clinical research. This should first be investigated in future studies before implementation. Moreover, a digital care platform generates a large amount of data and provides opportunities that should be explored in the future. Gathering and analyzing big data are growing areas in research, and they have the ability to provide insights in health care, for example, identifying patients at risk of treatment failure, adverse events, low medication compliance, and low guideline adherence. The use of big data can therefore improve guidelines and care for patients. The possibilities of big data should be explored in future research.

### Conclusion

We showed that a PAR approach is ideally suited for the improvement and scientific evaluation of eHealth innovations in daily clinical practice combined with the promotion of patient empowerment. The results suggest that the intensive involvement of patients in a PAR approach enables continuous improvement and evaluation of digital care platforms in daily clinical practice combined with the promotion of patient empowerment. Systematic iterative evaluation of users’ needs and wishes is required to keep care centered on patients and keep the innovation up-to-date and valuable for users. Since it is not possible to blind patients or withhold patients from using such effective digital tools, PAR is ideally suited to evaluate and improve the quality of their care.

## Appendix

Multimedia Appendix 1 StaRI (Standards for Reporting Implementation Studies) checklist.

Multimedia Appendix 2 SQUIRE 2.0 (Revised Standards for Quality Improvement Reporting Excellence) checklist.

Multimedia Appendix 3 Key points of the chronic myeloid leukemia guidelines.

Multimedia Appendix 4 Results from the first questionnaire after patients with chronic myeloid leukemia used the CMyLife platform for 3 months (n=54).

Multimedia Appendix 5 Results from the second questionnaire after patients with chronic myeloid leukemia used the CMyLife platform for 6 months (n=52).

## Abbreviations

CML: chronic myeloid leukemia
EMR: electronic medical record
HCP: health care professional
PAR: participatory action research
SUS: System Usability Scale
TKI: tyrosine kinase inhibitor

## Data Availability

The data sets used or analyzed during this study are available from the corresponding author on reasonable request.
