# Supplementary material for: Improvement, Implementation, and Evaluation of the CMyLife Digital Care Platform: Participatory Action Research Approach
Source: J Med Internet Res. 2023 Sep 15;25:e45259. doi: 10.2196/45259 (PMC10541637; doi:10.2196/45259)
Supplement: Multimedia Appendix 3 [file jmir_v25i1e45259_app3.docx]

**Multimedia Appendix 3.** Key points of the chronic myeloid leukemia guidelines.

| **Diagnosis** |  |
| --- | --- |
| Patients should get with complete diagnostic and molecular cytogenic workup at diagnosis | |
| **Treatment** |  |
| Patients should receive first-line TKI within 28 days after diagnosis | |
| **Follow-up** |  |
| Cytogenetic* and BCR-ABL (IS) monitoring at defined milestones and in case of milestone failure: | |
| - Performance of a mutation-analysis within 6 weeks - In presence of mutation: TKI switch - New BCR-ABL sampling within 2 months after failure | |
|  | At 3 months |
|  | At 6 months |
|  | At 12 months |
|  | At 24 months |
|  | At 36 months |
