# Supplementary material for: Improvement, Implementation, and Evaluation of the CMyLife Digital Care Platform: Participatory Action Research Approach
Source: J Med Internet Res. 2023 Sep 15;25:e45259. doi: 10.2196/45259 (PMC10541637; doi:10.2196/45259)
Supplement: Multimedia Appendix 4 [file jmir_v25i1e45259_app4.docx]

**Multimedia Appendix 4****.** Results from the first questionnaire after patients with chronic myeloid leukemia used the CMyLife platform for 3 months (n=54).

| **Instruction package**  Usability (very) usable *% (n)*  Language use (very) understandable  *% (n)*  Layout/design (very) appealing *% (n)*  Grade instruction package *mean (SD)*  Successfully installed the apps  Using instruction package *% (n)*  Without instruction package *% (n)*  With the help of the CMyLife-team *% (n)*  With the help of family/acquaintances *% (n)*    Contact CMyLife-team for installment (very) pleasant % *(n)*  Grade contact CMyLife-team *mean (SD)* | 79.6% (43)  81.5% (44)  40.7% (22)  7.1 (1.0)  29.6% (16)  14.8 % (8)  51.9% (28)  3.7% (2)  92.6% (50)  8.4 (0.9) |
| --- | --- |
| **Kick-off workshop**  Attended, yes *% (n)*  What did you think of the workshop (very) informative  *% (n)*  Grade starters workshop *mean (SD)* | 51.9% (28)  92.9% (26)  8.2 (0.9) |
| **Website**  Use website *% (n)*  Mainly uses question & answer *% (n)*  Mainly uses the forum *% (n)*  Mainly uses information *% (n)*  Mainly uses the news blog *% (n)*  Mainly uses video consultations *% (n)*  Findability information (very) good *% (n)*  Content (very) good *% (n)*  Usability (very) useful *% (n)*  Language use (very) understandable *% (n)*  layout/design (very) appealing *% (n)*  Recommend to other people with CML *% (n)*  Grade website *mean (SD)* | 70.4 (38)  34.2 (13)  44.7 (17)  73.7 (28)  23.7 (9)  2.6 (1)  89.5 (34)  100.0 (38)  89.5 (34)  97.4 (37)  71.1 (27)  100.0 (38)  7.7 (0.9) |
| **Medication app**  Use Medication app *% (n)*  Last used  Today *% (n)*  Last week *% (n)*  > a week , < month ago *% (n)*  > a month ago *% (n)*  How long have you been using the Medication app  Less than 1 month *% (n)*  Between 1-3 months *% (n)*  Between 3-6 months *% (n)*  At least 6 months *% (n)*  How often did you use the Medication app  Daily *% (n)*  Few times a week *% (n)*  Few times a month *% (n)*  Monthly *% (n)*  Top 5 most used components of the Medication app   1. Enter medication intake moments 2. Enter personal medication 3. CMyLife module on 4. Set medication reminders 5. Inventory management   Ease of use (very) good *% (n)*  Layout/design (very) appealing *% (n)*  Comprehensibility information (very) understandable *% (n)*  Misses side effects *% (n)*  Recommend to other people with CML *% (n)*  Use in preparation of consult with healthcare provider *% (n)*  Grade Medication app *mean (SD)* | 72.2 (39)  66.7 (26)  10.3 (4)  10.3 (4)  10.3 (4)  2.6 (1)  46.2 (18)  15.4 (6)  30.8 (12)  64.1 (25)  7.7 (3)  7.7 (3)  7.7 (3)  84.6 (33)  61.5 (24)  41.0 (16)  41.0 (16)  35.9 (14)  71.8 (28)  48.7 (19)  74.4 (29)  12.8 (5)  76.9 (30)  23.1 (9)  7.6 (1.3) |
