# Supplementary material for: Improvement, Implementation, and Evaluation of the CMyLife Digital Care Platform: Participatory Action Research Approach
Source: J Med Internet Res. 2023 Sep 15;25:e45259. doi: 10.2196/45259 (PMC10541637; doi:10.2196/45259)
Supplement: Multimedia Appendix 5 [file jmir_v25i1e45259_app5.docx]

**Multimedia Appendix 5.** Results from the second questionnaire after patients with chronic myeloid leukemia used the CMyLife platform for 6 months (n=52).

| **Delivered CML medication at home**  No *%(n)*  Yes, via the Medication app  *%(n)*  Yes, directly via the pharmacy *%(n)*  Yes, other *%(n)* | 53.8 (28)  11.5 (6)  23.1 (12)  15.4 (8) |
| --- | --- |
| **Where was your blood drawn in the past 6 months?**  In hospital of treatment *%(n)*  At my own GP practice *%(n)*  At a consultation hours supporting GP diagnostic center *%(n)*  At the thrombosis service *%(n)*  At home *%(n)*  No blood was drawn in the past 6 months *%(n)*  Other *%(n)* | 82.7 (43)  3.8 (2)  1.9 (1)  3.8 (2)  0.0 (0)  0.0 (0)  11.5 (6) |
| **Did you use CMyLife in the past 6 months?**  Yes, the website *%(n)*  Forum *%(n)*  Question & answer *%(n)*  News blog *%(n)*  Information about CML *%(n)*  other *%(n)*  Yes, the Medication app *%(n)*  Yes, the Guideline app *%(n)*  Yes, the personal health environment *%(n)*  Yes, other *%(n)*  No, not yet *%(n)* | 69.2 (36)  52.8 (19)  11.1 (4)  58.3 (21)  86.1 (31)  2.8 (1)  69.2 (36)  46.2 (24)  63.5 (33)  1.9 (1)  5.8 (3) |
| **Features of CMyLife that influence my knowledge about CML and its treatment**  Information on the website *%(n)*  Forum *%(n)*  Question & answer *%(n)*  News blog *%(n)*  Information about CML *%(n)*  Other *%(n)*  The Medication app *%(n)*  The Guideline app *%(n)*  The personal health environment *%(n)*  None of the above *%(n)*  Other *%(n)* | 76.9 (40)  32.5 (13)  7.5 (3)  50.0 (20)  85.0 (34)  5.0 (2)  25.0 (13)  17.3 (9)  25.0 (13)  13.5 (7)  1.9 (1) |
| **Features of CMyLife with the most influence on knowledge**  Information on the website (sum score)  The Medication app (sum score)  The personal health environment (sum score)  The guideline app (sum score) | 102  61  60  44 |
| **Using CMyLife gave me**  More control over my CML *%(n)*  Less control over my CML *%(n)*  No difference *%(n)* | 26.9 (14)  1.9 (1)  61.5 (32) |
| **Features of CMyLife that influence the degree of control over CML**  Information on the website *%(n)*  Forum *%(n)*  Question & answer *%(n)*  News blog *%(n)*  Information about CML *%(n)*  Other *%(n)*  The Medication app*%(n)*  The Guideline app *%(n)*  The personal health environment *%(n)*  None of the above *%(n)*  Other *%(n)* | 23.1 (12)  41.7 (5)  8.3 (1)  75.0 (9)  91.7 (11)  0.0 (0)  13.5 (7)  11.5 (6)  15.4 (8)  0.0 (0)  0.0 (0) |
| **Features of CMyLife with the most influence on degree of control over CML**  Information on the website (sum score)  The Medication app (sum score)  Het PGO (Persoonlijke Gezondheidsomgeving) (sum score)  The guideline app (sum score) | 24  20  18  11 |
| **Features of CMyLife that influence insight in side effects/complaints**  Information on the website *%(n)*  Forum *%(n)*  Question & answer *%(n)*  News blog *%(n)*  Information about CML *%(n)*  Other *%(n)*  The Medication app*%(n)*  The Guideline app *%(n)*  The personal health environment *%(n)*  None of the above *%(n)*  Other *%(n)* | 51.9 (27)  44.4 (12)  11.1 (3)  40.7 (11)  74.1 (20)  7.4 (2)  23.1 (12)  15.4 (8)  21.2 (11)  21.2 (11)  11.5 (6) |
| **Features of CMyLife with the most influence on insight in side effects/complaints**  Information on the website (sum score)  The personal health environment (sum score)  The Medication app (sum score)  The guideline app (sum score) | 79  55  48  43 |
| **System Usability Scale Medication app** *mean (min-max)* | 65.3 (30.0-100.0) |
| **Personal health environment**  Use Personal health environment *%(n)*  To enter BCR-ABL1 value *%(n)*  To enter medication *%(n)*  To Log side effects/symptoms *%(n)*  To keep a log *%(n)*  To enter measurements *%(n)*  Link with other apps, medication app and the guideline app *%(n)* | 65.4 (34)  82.4 (28)  44.1 (15)  41.2 (14)  8.8 (3)  17.6 (6)  11.8 (4) |
| **Did you use the personal health environment to give others insight in your health data?**  No *%(n)*  Yes *%(n)* | 57.7 (30)  5.8 (3) |
| **Guideline app**  Use Guideline app *%(n)*  How long have you been using the Guideline app  Less than 1 month *%(n)*  Between 1-3 months *%(n)*  Between 3-6 months *%(n)*  At least 6 months *%(n)*  How often did you use the Guideline app?  Daily *%(n)*  Few times a week *%(n)*  Weekly *%(n)*  Few times a month *%(n)*  Monthly *%(n)*  Other *%(n)*  Recommend to other people with CML *% (n)*  Use in preparation of consult with healthcare provider *% (n)*  Grade Guideline app *mean (SD)* | 48.1 (25)  4.0 (1)  12.0 (3)  56.0 (14)  16.0 (4)  0.0 (0)  8.0 (2)  8.0 (2)  28.0 (7)  36.0 (9)  20.0 (5)  84.0 (21)  32.0 (8)  7.0 (1.2) |
| **System Usability Scale Guideline app** *mean (min-max)* | 60.0 (37.5-97.5) |
